# Evaluating Nutrient Composition in Surplus Food Redistribution Programs: Evidence From Yogyakarta, Indonesia

**DOI:** 10.1002/fsn3.72501

**Published:** 2026-10-25

**Authors:** Marina Hardiyanti, Gyula Kasza, Ika Kurniati, Widya Satya Nugraha, David Szakos

**Affiliations:** ^1^ Jozsef Farkas Doctoral Program in Food Science Hungarian University of Agriculture and Life Sciences Budapest Hungary; ^2^ Department of Nutrition Health, Faculty of Medicine, Public Health and Nursing Universitas Gadjah Mada Yogyakarta Indonesia; ^3^ Department of Applied Food Science, Institute of Food Chain Science University of Veterinary Medicine Budapest Budapest Hungary; ^4^ Berbagi Bites Jogja Food Rescue Organization Yogyakarta Indonesia; ^5^ Department of Agricultural Socio‐Economics, Faculty of Agriculture Universitas Gadjah Mada Yogyakarta Indonesia

**Keywords:** food donation, food rescue, food waste prevention, nutrition evaluation, surplus food

## Abstract

Food rescue organizations act as hubs for food services, redistributing surplus food from their operations to recipients in need of food assistance to prevent food waste and reduce hunger. Ensuring the food is nutritious is the priority; however, this aspect is sometimes neglected. This study aimed to estimate the nutritional content and food group of redistributed surplus food against dietary recommendations in Indonesia. Data on the quantities, menus, and types of surplus food managed by the Food Rescue Organization were collected from July 2024 to October 2025 in Yogyakarta, Indonesia. We estimated nutritional content by calculating macronutrients using the Indonesian Food Composition Table (TKPI) database. Among all the redistributed food (*n* = 156 donation meals), the amounts of energy, total fat, and carbohydrate in all population groups did not meet 30% of the recommended dietary allowance (RDA) per meal, partly reflecting that some meals consisted of a single donated dish rather than a full combination of foods. Significant differences across donors were observed for protein (*p* < 0.001), total fat (*p* < 0.05), and carbohydrate (*p* < 0.05); however, the variance in the number of recorded meals across donors should be considered. Although the redistributed surplus food comes from various food groups, the dominant sources are grains and cereals and animal‐based protein. At the same time, high‐fiber food groups such as fruits, plant‐based proteins, and beans are in short supply. This study highlights the importance of improving nutrition in food rescue organizations' operations.

## Graphical abstract

This study estimated the nutritional content and food groups of surplus food redistributed by a food rescue organization in Yogyakarta, Indonesia (156 meals, July 2024—October 2025), and compared them with Indonesian dietary recommendations. Macronutrient content was calculated using the Indonesian Food Composition Table (TKPI). Energy, total fat, and carbohydrate did not reach 30% of the recommended dietary allowance (RDA) per meal in any population group, partly because some meals were a single donated dish rather than a full combination of foods. Protein (*p*< 0.001), total fat (*p*< 0.05), and carbohydrate (*p*< 0.05) differed significantly across donors. The food was drawn from various food groups, but grains and cereals and animal‐based protein dominated, while high‐fiber foods such as fruits, plant‐based proteins, and beans were in short supply. The findings suggest that food rescue organizations should improve the nutritional quality of their operations.

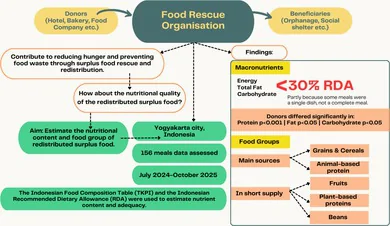

## 1 Introduction

Prioritizing halving global food waste by 2030 is a global concern and one of the United Nations Sustainable Development Goals UNEP (2024). Food waste in Indonesia, according to the latest data from the national information system on waste management in 2024, accounted for 38.29% of total waste, equivalent to 14,286,669 tons/year (SIPSN 2025). In Yogyakarta, food waste data accounted for 50.05%, equivalent to 354,533 tons/year or 971 tons/day (SIPSN 2025). The hotel, restaurant, and cafeteria (HORECA) sector accounts for varying proportions of total food waste across countries. For instance, in the European Union, food waste from the HORECA sector accounts for approximately 14 kg/capita, while in Ireland, Cyprus, and Malta, the contribution from the HORECA sector exceeds 30 kg/capita (Eurostat 2024). Findings from Sweden showed that food waste from HORECA sector ranged from 50.1 ± 9.4 g/portion to 192 ± 31 g/portion (Malefors et al. 2019). Moreover, findings in Finnish food services range from around 102 g/portion to 189 g/portion, while the other study in fast‐service and limited‐service restaurants ranged from 17 to 60 g/day/meal (McAdams et al. 2019; Silvennoinen et al. 2015). In Indonesia, edible food waste from the HORECA sector is equivalent to approximately 2,9 tons/day (Bappenas 2024; UNEP 2021). As the quantity suggests, it may seem like an insignificant amount; however, the food being wasted may be valuable to people experiencing food insecurity. Furthermore, the avoidable food waste produced continues to contribute to carbon emissions and affect the environment (Lins et al. 2021; Sigala et al. 2025). Commercial food services, such as HORECA and food companies, produce surplus food in their daily operations (Lins et al. 2021; Silvennoinen et al. 2015). In this case, food service operations are responsible for ensuring that their surplus food is well managed to prevent waste (Lins et al. 2021; McAdams et al. 2019; Sigala et al. 2025; Silvennoinen et al. 2015).

Rescuing surplus food means rescuing nutritious food from being wasted, addressing food insecurity, and helping reduce potential environmental impact (Hecht and Neff 2019; Thapa Karki et al. 2021; Torres Arroyo et al. 2024). Food rescue involves saving food for human consumption, recovering it, and redistributing it to groups that need assistance to provide food (Hecht and Neff 2019; Torres Arroyo et al. 2024). It is commonly seen as the social and business solution when surplus food in the food industry becomes a major problem (Mourad 2016; Torres Arroyo et al. 2024). In Indonesia, where the level of hunger is categorized as moderate, with a hunger index score of 14.6 (Global Hunger Index 2025), food donation or food charity organizations are growing as community initiatives to prevent food waste and contribute to reducing hunger and malnutrition (Bierwagen and Gonçalves‐Dias 2021; Clair et al. 2020; Putri et al. 2025; Tse and Tarasuk 2008). Food banks, food rescue, and related organizations serve as places to store a variety of rescued and donated food items from donor organizations and redistribute them to local communities or vulnerable groups in need of food support (An et al. 2019; Putri et al. 2025). To provide those services, food banks or charitable organizations face many challenges, such as the large quantity and variability of food items, personnel and technological issues, data management, and inventory management (Akkerman et al. 2023; Alkaabneh et al. 2021; Ata et al. 2019; Ding et al. 2023; Stauffer et al. 2022). Food donations are well‐intentioned; however, in practice, the people running the program lack knowledge of nutrition and health (Ravikumar‐Grant et al. 2023; Wetherill et al. 2019). Highlighting that the nutritional aspect of food donation is important, but the implementation is not prioritized, with the priority being to save as much as possible (quantity) rather than the quality (nutritional content and balanced food groups/variety of foods) (Irwin et al. 2007; Li et al. 2009; Mitchell and Prescott 2023; Nogueira et al. 2021; Verpy et al. 2003).

Although there is no agreement on the amount of food provided in the food donation program, it is hoped that food provision should be in good condition, consist of a variety of food groups, and contribute to meeting a third of the macronutrient needs for one meal (Mossenson et al. 2024; Simmet et al. 2017b; Zailani et al. 2023). Macronutrients such as energy, protein, fat, and carbohydrates are essential for human health (National Research Council (US) 1989; World Health Organization 2023b; World Health Organization 2023a). Not only macronutrient content but also food groups are important to consider, as they reflect the quality of the food consumed, which affects health outcomes and dietary behavior (Mossenson et al. 2024; Simmet et al. 2017a). According to guidelines by the Ministry of Health of Indonesia, the recommended dietary allowance amount of food consumed daily is equivalent to 300–400 g of vegetables and fruits for children and 400–600 g for adolescents and adults; 70–140 g of animal‐based protein, 100–200 g of plant‐based protein; and a variety of tubers, whole grains, and pulses that can meet an average calorie intake of 1850 kcal for children, 2300 kcal for adolescents, and 2258 kcal for adults. Additionally, the recommended proportions for protein are 18%–20%, fat 20%–25%, and carbohydrates 55%–65% of daily calories (Kementerian Kesehatan Republik Indonesia 2014). The intention of the food provision is not only to focus on nutrients but also to follow balanced dietary guidelines, ensuring that the food is varied and nutrient‐dense rather than calorie‐dense (Gombi‐Vaca et al. 2023; Levi et al. 2022; Nguyen et al. 2023; Roth et al. 2024).

To ensure redistributed surplus food is safe and healthy, a food rescue organization needs a framework to draft regulations on the quality of food it receives from donors. To build a foundation, the organization needs to understand the quality of the food it receives from donors, so this study aimed to assess the nutritional adequacy and variety of food groups in the surplus food redistributed by the food rescue organization. This study may provide a foundation for the food donation organization's recommendations on the nutritional adequacy of its food inventory and suggest ways food donors can improve the nutritional quality of their donations.

## 2 Methods

### 2.1 Organizational Profile

This cross‐sectional study collected data on surplus food rescue carried out by the Berbagi Bites Jogja Organization. Berbagi Bites Jogja is an independent food rescue organization that has been operating since July 2024, rescuing surplus food from commercial and non‐commercial food services, such as hotels, restaurants, caterers, bakeries, and cafes, and redistributing it to recipients across the Special Region of Yogyakarta, Indonesia. The recipients are mainly underprivileged university students living around the organization's office, and the program has since expanded to include orphanages, social shelters, elderly homes, and the general community experiencing food insecurity.

### 2.2 Data Collection and Analytical Unit

In this study, data were collected from eight food donors, which are 5 hotels, two bakeries, and one food factory, from July 2024 to October 2025. The donated food varies as listed in Table 1. This study extracts data from the organization's system. The available data consist of the name of the donor institutions, the type of donor institutions, the name of the recipients (can be individual names, orphanages' names, shelters' names), the date, the month, and the year of rescuing food, the name of the dishes, the amount of the food (in grams), and the number of recipients. The reported data are complete for all food collected during the reporting period. However, we excluded some data from the analysis because the information on the donated food was incomplete. For example, the data consist only of the food name, with no information on quantity or detailed ingredients, so the nutrients cannot be estimated.

**TABLE 1:** Description of food rescued from different food donors.

Food donors	Menu	Food groups
A	1. Fried rice	Grains and cereals
	2. Fried noodle	
	3. Fried thin rice‐noodle	
	4. Pasta	
	5. Fish stew	Animal protein
	6. Fried chicken	
	7. Butter chicken	
	8. Black pepper corned beef	
	9. Green vegetables sauteed	Vegetable
	10. Spicy‐stir fry eggplant	
B	1. Muskmelon	Fruit
	2. Watermelon	
	3. Papaya	
	4. Turmeric steamed rice	Grains and cereals
	5. Kwetiaw (Stir fry thick rice noodles)	
	6. Steamed white rice	
	7. Potato	
	8. White bread	
	9. Poridge	
	10. Porridge condiments	Additional
	11. Chips	
	12. Peanut sauce	Beans&nuts
	13. Nuts	
	14. Fritters	Additional
	15. Fried chicken	Animal protein
	16. Stir‐fry tofu and chicken with sweet soy sauce	Mixed‐based protein
	17. Stir‐fry tempeh (fermented soy)	Plant protein
	18. Red‐beans soup	Beans&Nuts
	19. Sautee green beans	Vegetable
	20. Stir‐fry assorted vegetables (Cap cai)	
	21. Assorted vegetables soup with coconut milk (Lodeh)	
	22. Chili paste	Additional
	23. Cakes	Sweets
**C**	1. Steamed white rice	Grains and cereals
	2. Fried noodle	
	3. Macaroni schotel	
	4. Fried egg	Animal protein
	5. Fried chicken	
	6. Chips	Additional
	7. Chili paste	
	8. Pudding	Sweets
	9. Cakes	
	10. Chocolate muffin	
D	1. Mixed rice and side dishes	Grains and cereals
	2. Fried noodle	
	3. Sweet ice tea	Sweets
E	1. Burger	Animal protein
	2. Chicken patty	
	3. Crispy fried chicken	
	4. Chicken nugget	
	5. Chicken karaage	
	6. Chicken meatball	
	7. Beef patty	
F & G	Bakery products	Grains and cereals
H	1. Fried rice	Grains and cereals
	2. Fried noodle	
	3. Sushi	
	4. Sandwich	
	5. Pasta	
	6. Crispy fried chicken	Animal protein
	7. Spicy stir‐fried egg	
	8. Stir‐fry assorted vegetables (Cap cai)	Vegetable
	9. Chicken spinach sauteed	Animal protein and vegetable
	10. Fritters	Additional

The total number of meals analyzed is 156 in total, distributed across donors as follows: Donor A *n* = 3; Donor B *n* = 1; Donor C *n* = 1; Donor D *n* = 2; Donor E *n* = 98; Donor F *n* = 45; Donor G *n* = 3; Donor H *n* = 3. The frequency of recorded data varied by months and donors; there were months with up to 10 donation events and months with fewer. The number of donation events also varied by donor, with some donors donating only once and others more frequently. Consequently, the number of meals excluded from analysis due to incomplete information also varied irregularly across months and donors, rather than following a consistent pattern. This irregularity in data collection frequency is addressed further in the study's limitations. The amount of food recorded in the organization's system reflects an estimated per serving portion weight, estimated by the organization's staff and volunteers at the point of distribution rather than measured against a donor‐supplied recipe or standard reference. This aspect of the methodology is also addressed further in the study's limitations.

The organization's database treated each donation event as a single analytical unit. A donation event refers to one instance of food donated by a donor and distributed by the food rescue organization to a recipient group on a given date. It could consist of either a single dish or multiple dishes distributed together on that occasion. Where a donation event included multiple dishes, we summed the nutrient content across all dishes. We divided by the number of recipients, representing the combined food received by one recipient at that donation event, defined as one meal. Where a donation event consisted of a single dish, the nutrient content of that dish, divided by the number of recipients, was treated as one meal. No additional meal construction or portion standardization was applied beyond this; the analysis reflects the food as it was distributed.

### 2.3 Food and Nutrient Estimation

A dish's name can also be used to estimate its macronutrient content. The authors identified the dishes and translated the calorie, protein, total fat, and carbohydrate content from the Indonesian Food Composition Table (Kementerian Kesehatan RI, 2018), published by the Ministry of Health of Indonesia. TKPI provides nutrient values at both the dish level (prepared or cooked dishes) and the ingredient level (individual food items). If the dish was available directly in TKPI, we used that value. If the dish name is not included in the TKPI table, the macronutrient composition is based on foods with similar ingredients or cooking techniques, identified from ingredients recognizable in the dish name. For example, the dish names include fried noodles, fried rice noodles, and fried thin rice noodles, but the TKPI includes only fried noodles. For the remaining noodle dishes, the estimate uses fried noodles.

The amount of food recorded in the organization's system showed an estimated per serving portion weight, estimated by volunteers in charge at the point of distribution, rather than measured against a donor supplied recipe or standard reference. Similarly, for bakery products and packaged foods, the recorded weight sometimes included non‐edible packaging, which may have introduced measurement error. These constraints are addressed further in the limitations section.

### 2.4 Food Group Classification

From the names of the dishes, the author identified and categorized the food into nine food groups: grains and cereals; animal‐based protein; vegetables; fruit; plant‐based protein; mixed‐based protein; beans & nuts; sweets; and additional. This classification was based on the Household Dietary Diversity Score (HDDS) (FAO, 2021) food group framework by the Food and Agriculture Organization, combined with study‐specific groupings developed to reflect the composite nature of donated dishes containing both plant‐ and animal‐based ingredients, categorized as mixed‐based protein.

Grains and cereals include dishes such as rice, noodles, pasta, rice noodles, bread, and porridge. Animal protein consists of chicken, fish, beef, eggs, corned beef, processed chicken meat, and processed beef meat. Vegetables consist of assorted vegetables, mostly cooked in sauté, stir‐fry, or clear soup styles. Fruit is available sliced, while additional items include assorted condiments, sauces, sprinkles, and toppings for other main dishes. Beans and nuts are mostly cooked in soups and as peanut sauces. Mixed‐based protein is found in a dish with both plant‐based and animal‐based protein ingredients found in the dishes. For the plant protein, the main ingredient is fermented tempeh, and for the sweets, they are bakery products and confectionery.

### 2.5 Adequacy Criteria

The adequacy of the macronutrients in the dishes is compared to Angka Kecukupan Gizi (AKG) or RDA for the Indonesian population (Kemenkes RI, 2019), and categorized into three categories: child, adolescent, and adult populations, and presented as a percentage. We calculated the RDA values in Table 2 by averaging the sex‐specific recommendations from the AKG 2019 for each broad age category. For children, we averaged values across males and females aged 7–12 years. For adolescents, values were averaged across males and females aged 13–18 years. For adults, values were averaged across males and females aged 19–64 years. We applied the RDA categories across this broader range because the organization's data did not include beneficiaries' specific age and sex information. Recipients' data record age only as primary school‐aged children, middle‐ and high school‐aged adolescents, and adults. This constraint on the data availability is addressed further in the limitations.

**TABLE 2:** Recommended dietary allowance based on Indonesian population standard (AKG 2019).

Population group	Recommended dietary allowance (RDA)			
	Energy (kcal)	Protein (gram)	Total fat (gram)	Carbohydrate (gram)
Child	1850	49	62	277
Adolescent	2300	69	76	338
Adult	2258	63	64	361

Although there is no agreement on the amount of food provided in the food donation program, it is hoped that food provision should be in good condition, consist of a variety of food groups, and contribute to meeting a minimum of one‐third (30%) of daily macronutrient needs (Mossenson et al. 2024; Simmet et al. 2017a; Zailani et al. 2023).

### 2.6 Statistical Analysis

Microsoft Excel for Mac version 16.78 was used to create data tabulation before statistical analysis in SPSS. IBM SPSS Statistics for Mac Version 29.0.2.0 was used to analyze statistical data. To compare proportions of meals meeting the 30% RDA across age groups and donors, and to compare food groups of donated food across food donors, the Likelihood Ratio Chi‐square test was used instead of the Pearson Chi‐square, as several cells had expected frequencies below the conventional threshold of 5. For pairwise comparisons among donors when the overall test was significant, we used Fisher's Exact Test for 2 × 2 tables, with Bonferroni correction to control for multiple comparisons. We used a *p*‐value threshold of < 0.05 to determine statistical significance.

## 3 Results

Table 2 shows four primary nutrient contents: energy, protein, total fat, and carbohydrate in three age group categories: child, adolescent, and adult, according to the RDA used in Indonesia. Daily energy recommendations are approximately 1850 kcal for children and 2300 kcal, the highest recommendation, for adolescents. Recommended protein consumption amounts for children, adolescents, and adults are 49 g, 69 g, and 63 g, respectively. The total fat dietary allowance for adolescents is the highest, while it is the lowest for children. Furthermore, for carbohydrates, the recommended value is highest in the adult population group with 361 g.

Table 3 shows the estimated nutritional value of redistributed food by age group. The mean energy content per meal ranged from 202 kcal (adolescent) to 338 kcal (adult). The protein content of the donated food met the 30% threshold on average for children (46.9% of RDA) and adults (37.9% of RDA), but not for adolescents (17.9% of RDA). Total fat and carbohydrate content were consistently below 30% of the RDA across all age groups.

**TABLE 3:** Nutrition value estimation from the redistributed surplus food data.

Macronutrients	*n*	Mean (95% CI)	SD	Median (min‐max)	% of Daily RDA
Child					
Energy (kcal)	30	299 (234–365)	176	235 (123–827)	16.1
Protein (gram)	30	23.0 (16.0–30.0)	18.8	14.5 (9.0–85.0)	46.9
Total Fat (gram)	30	13.8 (10.4–17.3)	9.2	10.0 (1.0–43.0)	25.3
Carbohydrate (gram)	30	19.6 (9.9–29.3)	26.1	8.0 (0.0–121.0)	9.0
Adolescent					
Energy (kcal)	21	202 (148–257)	120	188 (20–582)	8.6
Protein (gram)	21	13.3 (8.4–18.2)	10.7	11.0 (2.0–47.0)	17.9
Total Fat (gram)	21	8.3 (5.5–11.0)	6.1	8.0 (1.0–24.0)	10.4
Carbohydrate (gram)	21	18.4 (11.0–25.8)	16.2	16.0 (0.0–49.0)	5.1
Adult					
Energy (kcal)	105	338 (290–386)	246	302 (70–2191)	16.3
Protein (gram)	105	21.4 (18.2–24.6)	16.7	15.0 (3.0–90.0)	37.9
Total Fat (gram)	105	9.8 (7.9–11.6)	9.6	6.0 (1.0–45.0)	15.1
Carbohydrate (gram)	105	39.5 (28.7–50.3)	55.9	27.0 (0.0–442.0)	12.3

A chi‐square test evaluated the adequacy of macronutrient intake at ≥ 30% across three population standards. From the statistical analysis, there is no significant association between the population group and meeting 30% of the recommended intake for all macronutrients, indicating that across all population groups, there is no significant difference in the adequacy of 30% macronutrient intake (Table 4a).

**TABLE 4a:** Comparison of the nutritional value that fulfills 30% of RDA in different age groups.

Nutrient	Child (*n* = 30)	Adolescent (*n* = 21)	Adult (*n* = 105)	*p* *
Energy	10.0%	0.0%	4.8%	0.269
Protein	50.0%	23.8%	40.0%	0.170
Total Fat	16.7%	4.8%	19.0%	0.276
Carbohydrate	3.3%	0.0%	5.7%	0.485

* *p*‐value from Likelihood Ration Chi‐square test.

A similar statistical test was performed to determine the differences in nutritional value that met 30% of the RDA among eight food donors. The results indicate that there were no significant differences in energy adequacy across donors. However, statistically significant differences were observed for protein (*p* < 0.001), total fat (*p* = 0.006), and carbohydrate (*p* = 0.008). Pairwise comparisons for protein with Bonferroni correction (8 comparisons) revealed that Donor A had a significantly higher proportion of protein‐adequate meals compared to Donor F; however this comparison is based on only three meals from Donor A and should be interpreted with caution. No other pairwise comparisons were significant after correction (Table 4b). As donation events varied in how many dishes were combined per event, with Donor A's meals each summing four to five dishes, compared to single‐dish meals for donors such as F and G, donor‐level adequacy differences may partly reflect this structural variation rather than nutritional quality alone, and this is addressed further in the study's limitation.

**TABLE 4b:** Comparison of the nutritional value that fulfills 30% of RDA in different food donors.

Nutrient	A (*n* = 3)	B (*n* = 1)	C (*n* = 1)	D (*n* = 2)	E (*n* = 98)	*F* (*n* = 45)	G (*n* = 3)	H (*n* = 3)	*p* *
Energy	33.3%	0%	0%	0%	4.1%	6.7%	0%	0%	0.553
Protein	100%^**(a)**^	0%	0%	50%	53.1%	11.1%^**(b)**^	0%	33.3%	< 0.001*
Total Fat	66.7%	0%	0%	0%	23.5%	0%	0%	33.3%	0.006*
Carbohydrate	33.3%	0%	0%	0%	0%	13.3%	0%	0%	0.008*

*Note:* Superscript letters: pairwise comparisons using Fisher's Exact Test with Bonferroni correction (8 comparisons); donors sharing no superscript letter differ significantly at *p* < 0.05.

* Overall *p*‐value: Likelihood Ration Chi‐square test.

The redistributed surplus foods from the food donors were categorized into nine groups: grains and cereals, animal protein, vegetables, fruit, additional, beans & nuts, mixed‐based protein, plant protein, and sweets. Grains and cereals include dishes such as rice, noodles, pasta, rice noodles, bread, and porridge, and are mainly carbohydrate‐rich. Animal protein consists of chicken, fish, beef, eggs, corned beef, processed chicken meat, and processed beef meat. Vegetables consist of assorted vegetables that are mostly cooked in sauté, stir‐fry, or clear soup styles. Fruit is available as sliced fruit, while additional items include assorted condiments, sauces, sprinkles, and toppings for other main dishes. Beans and nuts are mostly cooked as soups and as peanut sauces mixed‐based protein found in a dish with both plant‐based and animal‐based protein ingredients in the dishes. For the plant protein, the main ingredient is fermented tempeh, and for the sweets, they are bakery products and confectionery (Table 1).

The variability of grain and cereal dishes and animal protein dishes is wider than that of other foods, resulting in uneven comparisons (Figure 1A). In addition, the food groups donated by each donor are also uneven; for example, Donor E mostly donated animal‐based protein, while Donor FG mostly donated grain‐type foods. Pairwise comparisons with Bonferroni correction of the donated food types are significantly different, especially between animal‐based and non‐animal‐based protein dishes for Donor E (*p* < 0.05) (Figure 1B).

**FIGURE 1A:**
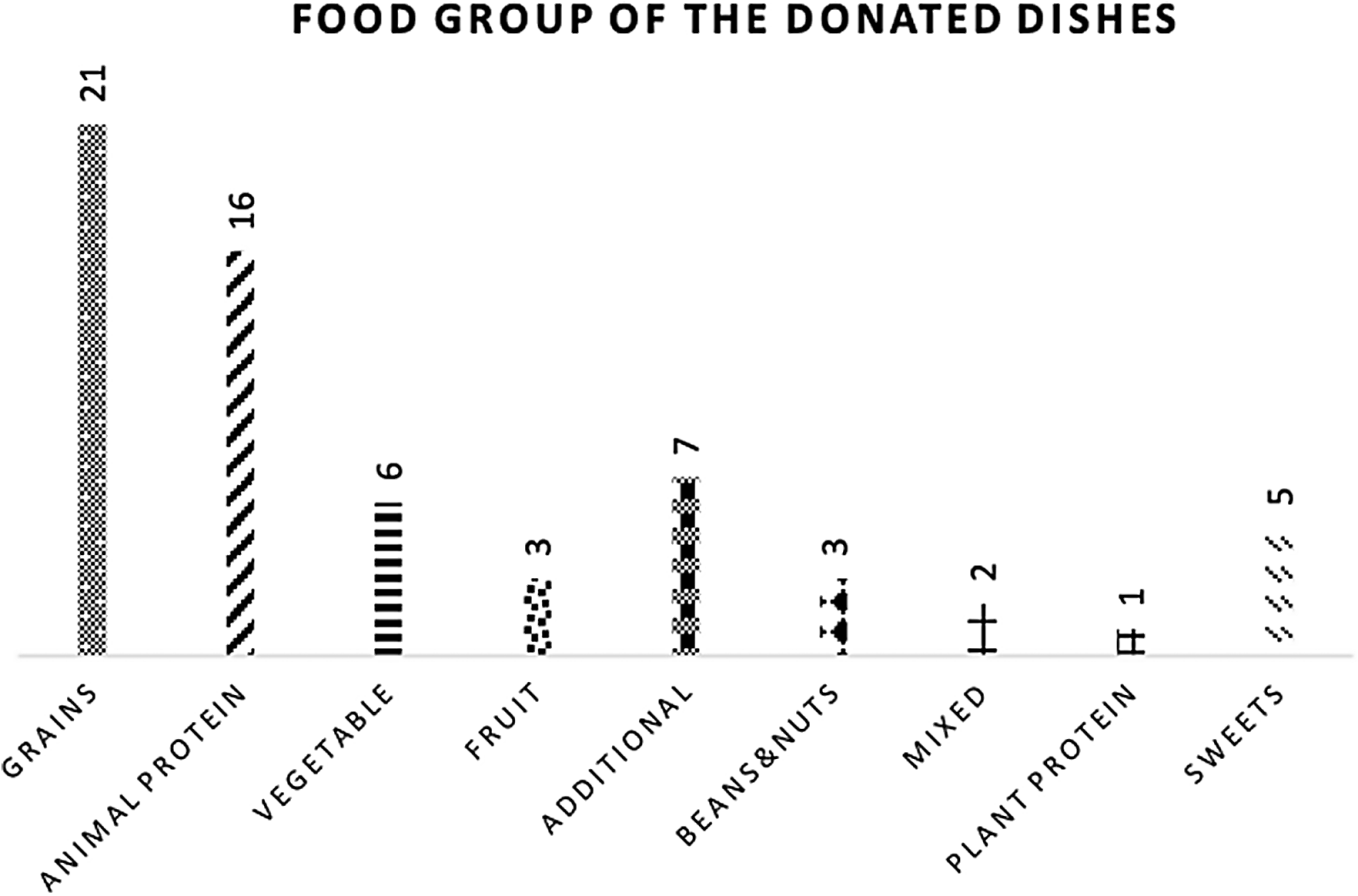
Variety of food rescued based on the food groups.

**FIGURE 1B:**
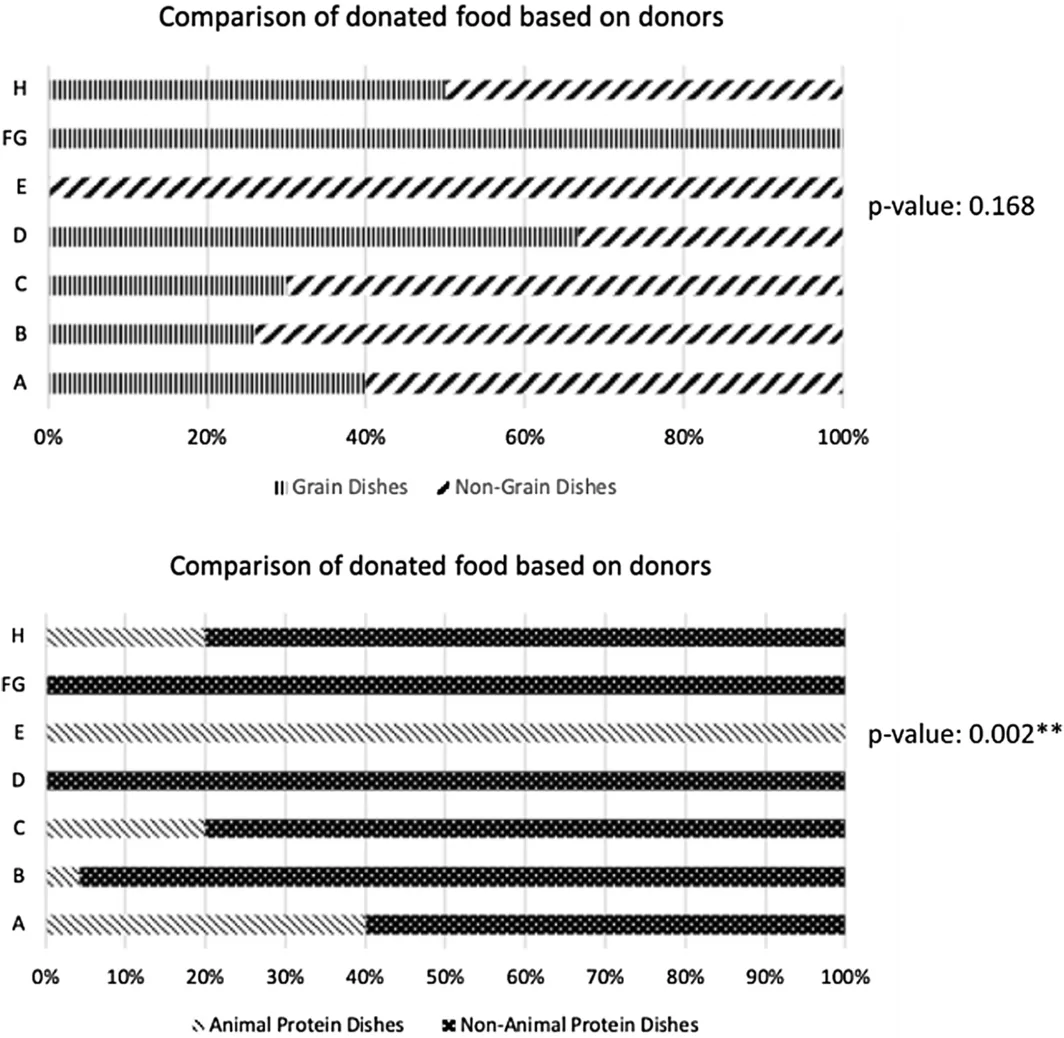
Comparison of donated food distribution across donors by food group. Fisher's Exact Test, *p* > 0.05 after Bonferroni correction. **p < 0.05.

## 4 Discussion

Aiming to address the food insecurity of the group that needs food assistance, while preventing food waste from surplus food production, food rescue organizations intend to ensure sufficient nutrition in their food provisions. However, this objective is not easy to achieve, as evidenced by the finding that the majority of macronutrients in all groups were below 30%. In this study, only protein adequacy met the 30% threshold on average for children and adults, while energy, total fat, and carbohydrate did not meet this standard. This finding is in line with the previous studies conducted in Canada (Irwin et al. 2007; Tse and Tarasuk 2008), the United Kingdom (Frost et al. 2016; Pelham‐Burn et al. 2014), and Portugal (Nogueira et al. 2021) that reported that the nutritional composition in food donation programs tends to be insufficient. Findings from a study in the United Kingdom (Pelham‐Burn et al. 2014) indicate that breakfast meals from charitable organizations for homeless people have an energy content of only 5.3%. In this study, the RDA for adolescents was higher than that for children and adults, reflecting the greater nutritional demands of this population during growth and development (National Research Council (US) 1989). Although the demand in this age group population is higher, in this study the observed adequacy for the adolescent population is the lowest in percentage fulfillment of energy, protein, total fat, and carbohydrate compared with children and adults (Table 3). At the same time, food redistribution received from the donor that was distributed to this group is limited, suggesting that its nutrient value may be inadequate precisely where physiological demand is highest. Other relevant studies reported various findings. Some findings show that food provision for adolescent groups in food assistance programs is imbalanced with their nutritional requirements (Li et al. 2009; Mitchell and Prescott 2023); however, other studies have found positive results in meeting nutrition recommendations (Mousa and Freeland‐Graves 2019; Nogueira et al. 2021; Vaiciurgis et al. 2024). A possible explanation for this difference relates to the type of surplus food and donor source involved. Unlike food bank or food pantry models that tend to rely on grocery, packaged, or fresh produce donations, the surplus food examined in this study comes from HORECA donors and consisted of ready‐to‐eat meals prepared for single servings or buffet, such as fried rice, noodles, and bakery products, rather than portioned food to meet a recipient's full nutrient requirement. This characteristic suggests that the type of donor and food source, not only organizational capacity, is an important factor influencing whether nutrition recommendations are met.

Investigating the fulfillment of 30% of energy, protein, total fat, and carbohydrate across three RDA categories of the population revealed no differences among the three population groups (Table 4a), indicating no association between the RDA population group and the fulfillment of 30% macronutrients. Hence, statistically significant differences were observed across donors for protein (*p* < 0.001), total fat (*p* = 0.006), and carbohydrate (*p* = 0.008), with Donor A having the highest proportion of protein‐adequate meals. Pairwise comparisons for protein with Bonferroni correction revealed that Donor A had a significantly higher proportion of protein‐adequate meals compared to Donor F. No other pairwise comparisons were significant after correction. This result should be interpreted with caution, as it is based on only three meals from Donor A. Each of Donor A's three donation events combined four to five dishes: donation Event 1 consisted of fried rice, fried chicken, fried noodles, and fish stew; Event 2 consisted of fried rice, fried chicken, green vegetable sautéed, fried thin rice noodle, and black pepper corned beef; and Event 3 consisted of fried noodles, spicy stir‐fry eggplant, butter chicken, fried rice, and pasta. Because meal‐level nutrient values were calculated as the sum across all dishes in a donation event, and because each of Donor A's three events included at least one substantial animal‐protein item such as fried chicken, corned beef, or butter chicken alongside grain‐based dishes, the combined nutrient totals for Donor A were consistently higher in protein than donors whose donation events typically consisted of a single dish (e.g., Donor F, G). This difference in how donation events were structured and recorded, and in the consistent presence of an animal‐protein item across Donor A's meals, rather than a general difference in donor nutritional quality, may account for a substantial part of the observed donor‐level variation in adequacy.

In Figure 1A, the variety and frequency of redistribution of grains and cereals, animal‐based protein, vegetable‐based protein, and mixed‐based protein food groups were high. In contrast, fruits, plant‐based protein, beans, and nuts were lower. This finding is supported by a previous study showing that fresh foods and fiber‐rich food groups are less frequently found in donated food (Irwin et al. 2007; Li et al. 2009; Mitchell and Prescott 2023; Verpy et al. 2003; Wingrove et al. 2017). Another study also found that, in general, the nutritional content of food provided by food pantries is inadequate and lacks fresh foods such as fruits and vegetables (Campbell et al. 2013; Gibson et al. 2022; Simmet et al. 2017b).

The surplus food redistributed in this study was dominated by ready‐to‐eat meals that volunteers in the food rescue organization partly reheated or recooked before delivering them to the recipients. Apart from ingredients, cooking methods also affect the nutritional value of foods. As shown in Table 1, the foods were mainly cooked by frying, either deep‐fried or pan‐seared, which may contribute to the additional fat content. In addition, some food donors donated surplus food that contained plenty of processed meat, such as nuggets and burger patties. Despite meat's protein content, processed meat is less desirable from a health perspective than fresh meat. This finding emphasizes the need for volunteers in food rescue, food pantries, and other relevant programs to enhance their knowledge of healthier food choices and their ability to use various cooking methods. Many studies report that challenges in food donation organizations are commonly related to nutrition knowledge, food choices, and cooking ability, as well as the lack of personnel and cooking and storage infrastructure (Hewitt et al. 2007; Hudak et al. 2022; Pelham‐Burn et al. 2014; Wetherill et al. 2019; Wicks et al. 2006; Yousey et al. 2007).

Basic nutrition knowledge may be relevant not only for the food rescue organization's staff but also for the food donors, the institutions that produce and donate surplus food. This study's findings can strengthen the foundation for food rescue organizations to develop organizational regulations for food donors to improve the quality of surplus donated food. Moreover, this finding can be used to educate food donors and recipients about the food they donate and receive, thereby improving recipients' health outcomes. For policy stakeholders at the national and regional levels, this finding may not be generalized. However, it shows that policy‐related food donation operations and nutrition‐related policies for food donation programs are needed. Overall, organizations and all actors involved in rescuing food work not only to prevent food waste and reduce hunger but also to support public health outcomes and build more sustainable organizations.

## 5 Limitation

This study only came from one food rescue organization; although its activities covered all areas in the Special Region of Yogyakarta Province, the generalizability may be limited. Several aspects of the data collection process should also be considered when interpreting these findings. The frequency of data collection varied by month and donors. As a result, the number of meals excluded from analysis due to incomplete information also varied irregularly across months and donors, rather than following a consistent pattern, meaning that donors or months with fewer recorded collection days may be underrepresented in the pooled nutrient estimates presented in this study. The number of donation events per donor was highly uneven (ranging from *n* = 1 to *n* = 98), which limits the reliability of donor‐level comparisons, particularly for donors with very few recorded events (B–D).

In addition, donation events varied considerably in how many dishes were bundled together, ranging from single‐dish events (e.g., Donor F, G) to events combining up to 23 dishes (Donor B). Because meal‐level nutrient values were calculated by summing across all dishes within a donation event, donors whose donation events typically involved more dishes produced higher per‐meal nutrient totals than donors whose events consisted of a single dish. As a result, donor‐level differences in adequacy reported in this study may partly reflect this structural difference in how donations were delivered and recorded, rather than genuine differences in the nutritional quality of what each donor provided. This should be considered when interpreting the statistically significant donor‐level comparisons reported above.

Some methods can be improved by measuring food amounts, actual age, and recipient gender, thereby improving the accuracy of RDA and nutrient estimates. In particular, the amount of food recorded in the organization's system reflects an estimated per‐serving portion weight, estimated by volunteers at the point of distribution rather than measured against a donor‐supplied recipe or standard reference, which may introduce measurement error into the reported nutrient values. Similarly, recipient records classified age only into broad categories: primary school‐aged for children, middle and high school‐aged for adolescents, and adult, rather than specific age, which limited the RDA comparison to three broad population groups rather than the finer age bands defined in AKG 2019. This may obscure meaningful variation in nutrient adequacy within each group, for example, between a middle school‐aged and high school‐aged recipient, both classified as adolescent.

In addition, the Indonesian Food Composition Table may be limited in estimating nutrient content because its food list is limited. Where a dish name had no direct match in TKPI, nutrient values were estimated using foods with similar ingredients or cooking techniques rather than the donor's actual recipe or nutrition labeling, which may introduce bias into the nutrient estimates, particularly for fat content, given that commercial preparation methods such as frying can vary considerably from the generic reference dish used for estimation.

The 30% RDA threshold used in this study assumes a complete, composed meal. Not all redistributed food met this definition, as some donations consisted of standalone food items, such as fruit or bread, rather than a full meal. Such items cannot meet 30% of the RDA regardless of their nutritional quality, which should be considered when interpreting the low adequacy percentages observed for some food groups and donors.

Finally, some aspects of the statistical analysis have their own limitations. To compare proportions of meals meeting the 30% RDA threshold, we used the Likelihood Ratio Chi‐square test for the overall analysis because Fisher's Exact Test is not available for tables larger than 2 × 2 in our SPSS version. To avoid inappropriate statistical results, we used Fisher's Exact Test for pairwise comparisons in 2 × 2 tables, with Bonferroni correction applied. Future analyses with these data may benefit from using Fisher's Exact Test for larger tables given the sparsity of some cells.

## 6 Implication of the Study

This study suggests that food rescue organizations can serve as important hubs connecting food businesses or donors that produce surplus with people experiencing food insecurity. Findings from this single organization case suggest that food rescue organizations may benefit from developing organizational policies on the composition and nutritional variability of donated food that balance the interests of donors, recipients, and the organization itself. Based on the estimated macronutrient composition of donated dishes in this study, ensuring adequate nutritional quality and availability of fresh foods appears to be a responsibility best shared between food rescue organizations and food donors, rather than resting with either party alone. These findings highlight the potential value in organizational guidelines that address the variability of donated food and suggest that food rescue organizations may consider encouraging donations of more nutrient‐dense food while limiting the proportion of food high in sugar, salt, and fat. Food donors and food businesses that regularly produce surplus food may similarly benefit from greater awareness of the estimated nutritional composition of their donations and its potential relevance to recipient health, rather than viewing food donation solely as a means of reducing organizational food waste.

This study also suggests that food donation organizations, public or private, social or faith‐based, may benefit from stronger collaboration to address shared operational barriers. As this study is based on data from a single organization in one region of Indonesia, the findings cannot be generalized nationally or to food rescue organizations more broadly. Nonetheless, they offer preliminary, context‐specific evidence on the volume, variety, and estimated nutritional adequacy of food redistributed through this organization, which may inform future research examining the broader contribution of food rescue organizations to food security and waste reduction. These findings may be of interest to regional leaders and policymakers considering practical approaches to support food donation operations and encourage donor practices aligned with nutritional and health considerations.

## 7 Conclusion

This study provides evidence that the nutritional quality of surplus food redistribution within the food rescue organization needs improvement; at least one meal meets 30% of the macronutrient recommendations, and healthier food sources can be increased. Food rescue organizations can positively contribute to sustainable development goals by preventing food waste, reducing hunger and food insecurity, and improving health outcomes; therefore, responsibility lies not only with the food rescue organization but also with all actors involved in its activities.

## Author Contributions

**Marina Hardiyanti:** conceptualization, methodology, data curation, visualization, writing – original draft. **Gyula Kasza:** methodology, writing – review and editing, supervision. **Ika Kurniati:** conceptualization, methodology, writing – review and editing, project administration. **Widya Satya Nugraha:** methodology, data curation, visualization, writing – review and editing. **David Szakos:** methodology, supervision, writing – review and editing.

## Funding

This study was funded by the “Hibah Dana Masyarakat Pengabdian Kepada Masyarakat Skema Terintegrasi 2025” with the registered number 5697/UN1/KU/KAP/PM/01.03/2025 from Community Service Funding Faculty of Medicine, Public Health, and Nursing, Universitas Gadjah Mada, Indonesia and the Janos Bolyai Research Scholarship of the Hungarian Academy of Sciences.

## Ethics Statement

This study does not involve any testing, direct treatment, or intervention on humans. The authors and data collection team ensure the confidentiality of the data. The involved organization gave consent and was fully aware of the data usage in this study.

## Conflicts of Interest

The authors of this study stated the following professional relationship, which may be considered a potential competing interest: Marina Hardiyanti and Ika Kurniati report having collaborated on a community service project with the Berbagi Bites Jogja Organization. As for the other authors, they declared no personal or institutional conflicts of interest that can influence this work.

## Data Availability

The data that support the findings of this study are available from the corresponding author upon reasonable request.
